# Micro-CT Anatomy of the Vertebral Column of the Luristan Newt (*Neurergus kaiseri*)

**DOI:** 10.1155/vmi/6958388

**Published:** 2025-06-04

**Authors:** Yasin Valizadeh, Mohammad Nasrolahzadeh Masouleh, Omid Zehtabvar, Saied Bokaie

**Affiliations:** ^1^Department of Veterinary Clinical Sciences, SR.C., Islamic Azad University, Tehran, Iran; ^2^Anatomy Sector, Department of Basic Sciences, Faculty of Veterinary Medicine, University of Tehran, Tehran, Iran; ^3^Epidemiology and Zoonosis Division, Department of Food Hygiene, Faculty of Veterinary Medicine, University of Tehran, Tehran, Iran

**Keywords:** Kaiser's mountain newt, micro-CT, morphology

## Abstract

The *Neurergus kaiseri* is one of the native and endangered species of the Salamandridae family, which is restricted to the mountainous habitats of southern Lorestan and northern Khuzestan. The presence of this species in the environment and the risks surrounding the extinction and health of this species make it necessary to produce basic anatomical information. The present study investigated the typical morphological characteristics of normal, mature, and healthy Luristan newt (*Neurergus kaiseri*) vertebral column using a micro-CT scan. The samples were entered into the micro-CT scan machine one by one in a ventral recumbency. The typical morphological characteristics of normal, mature, and healthy Luristan newt (*Neurergus kaiseri*) vertebral column were examined. It was specified that the Luristan newt (*Neurergus kaiseri*) has one cervical vertebra (atlas), 12 trunk vertebrae, one sacral vertebra, 3 caudosacral vertebrae, and 28 to 31 caudal vertebrae. This study presents a complete and precise description evaluation of Luristan newt (*Neurergus kaiseri*) vertebral column using a micro-CT scan. No specimen was killed, and anatomical studies were conducted through a micro-CT scan technique as an essential feature of the present study.

## 1. Introduction

Amphibians are a special group of vertebrates, that spend part of their lives in water and in a wet environment [1]. They represent a significant contribution to the diversity of vertebrate life on earth with more than 4000 species [2].

The vertebrate skeletal system and its elements are important in evolutionary biology. Vertebrate skeletons can be considered as a combination of apparently separate units (i.e., bones), which has attracted the attention of comparative anatomists [3].

In Iran, the suborder Salamandroidea is represented by a single family, Salamandridae, which includes three genera: *Triturus, Neurergus*, and *Salamandra*. The genus *Neurergus* has a relatively wide geographic distribution, ranging from the Zagros Mountains in western Iran to Iraq and southern Turkey. This genus comprises four species: *Neurergus strauchii*, *Neurergus crocatus*, *Neurergus microspilotus*, and *Neurergus kaiseri* [4].

Luristan newt (*Neurergus kaiseri*) is one of the newt species native to Iran, which lives in a limited area in the southern Zagros Mountain range. The breeding habitats of *Neurergus kaiseri* are upland streams and ponds that are often active in spring and are often temporary or seasonal. When ponds dry up during the spring and early summer, newts migrate from the ponds to the surrounding rugged mountains, which are covered with dry forests and scrubs; however, there is little information in this regard [5].

This species lives in rivers and high ponds in oak forests. This newt is classified as vulnerable (VU) on the International Union for Conservation of Nature (IUCN) red list of threatened species due to its small range, illegal trade, habitat loss and climate-induced drought [6]. It is also included in Appendix I of the Convention on International Trade in Endangered Species [7].

**Figure 1:**
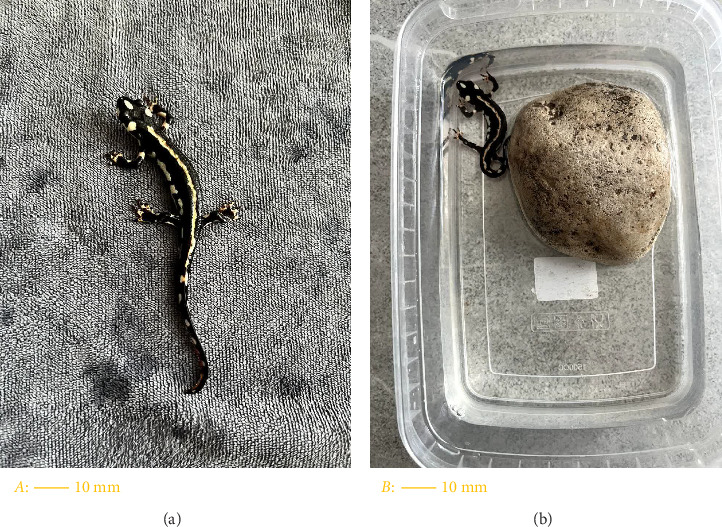
(a) Gross morphology of *Neurergus kaiseri*. (b) Newt transfer technique from habitat to experiment.

*Neurergus kaiseri* is distinguished from other species of Neurergus by black spots and an orange dorsal line [8] (Figure 1(a)).

There are few regional changes in the vertebral column of newts and the general assumption is that they occur less often. Worthington and Wake [9] identified the following five regions in the terrestrial newt: atlas (cervical vertebra), trunk vertebrae, sacrum, caudosacral vertebrae and caudal vertebrae.

Vertebral column consists of a series of distinct but repeated cartilaginous or bony elements. In lampreys, several small cartilaginous elements, neural arches and spines, are mounted on a prominent notochord, but the main body of the vertebrae is absent [10]. Ostracoderms rarely refer to the vertebral column, which may be because vertebrae are probably present but either boneless or weak [10].

CT scan is a three-dimensional x-ray imaging method that employs x-ray radiation at many angles around an axis and then uses a tomographic reconstruction pattern to produce serial transverse and closely spaced images [11].

In recent years, micro-CT has advanced in many research areas and is becoming a routine microscopic technique [12]. A number of technical advances in x-ray sources and x-ray imaging arrays have expanded the application of micro-CT. Although the term micro-CT is usually used for CT scanners with a voxel resolution of less than a millimeter, a more appropriate generic name is now micro-CT scanning [11].

The technologies used in micro-CT scanners are basically the same, but they differ in terms of the quality and resolution of the CT image, as well as the size of the three-dimensional image [11].

The purpose of this study is to investigate the structures of the vertebral column in the Luristan newt (*Neurergus kaiseri*) as an endangered species with other salamander species.

## 2. Materials and Methods

### 2.1. Animals

Considering the special protection status of this species and its national protection status, which is among the endangered species, to collect the samples, coordination and correspondence were made with the relevant organizations and their consent was obtained. Five adult male *Neurergus kaiseri* newts and five adult female *Neurergus kaiseri* newts were caught in the region during two time periods. The samples were sexed based on cloacal morphology. The male has a fleshy protuberance at the cranial end of the valve slit, while the female lacks a protuberance in this area [13]. To store and transfer the samples to the preclinical laboratory of Tehran University of Medical Sciences, plastic containers were used. Considering that the *Neurergus kaiseri* lives in reservoirs and ponds, these containers contained some cold water (temperature of about 17°C–20°C), and also a number of stones were placed outside the water to create a dry and humid environment. Therefore, the samples had access to both dry and water environments (Figure 1(b)).

### 2.2. Micro-CT Scan

**Figure 2:**
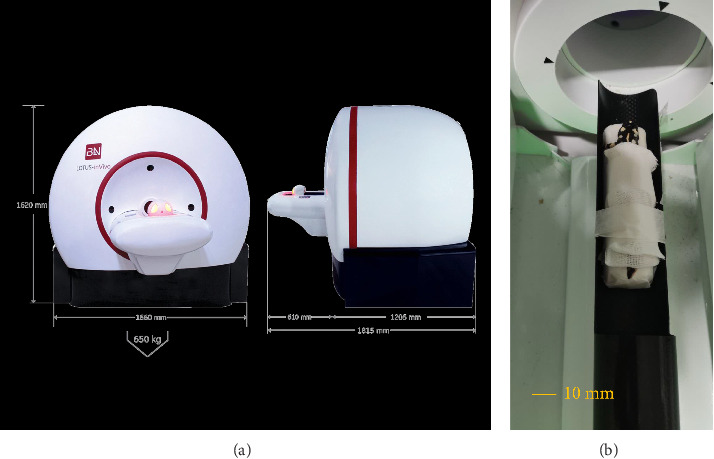
(a) The micro-CT scanner. (b) Positioning the newt prior the micro-CT scanning.

One day after being caught, the samples were transferred to the micro-CT scan Department of the Pre-clinical Laboratory of Tehran University of Medical Sciences. An effort was made to position the animal for the micro-CT scan in such a way that the body of the animal is stretched and the organs are also stretched, symmetrical. An attempt was also made to avoid laying organs on the body, if possible, in such a way that the body of the animal is completely parallel to the table. Tricaine methane sulphonate (MS-222) (10 mg/L of water) was used as an anesthetic pool and for temporary immobilization [14]. Afterwards, an adhesive tape was used to prevent the samples from changing their position during micro-CT scanning. To prevent damage caused by adhesive tape on the skin of the samples, first, a tampon layer was placed on the body of the animal, and then they were bound by the adhesive tape (Figure 2). All samples were placed in the ventral recumbency on the table of the micro-CT scanner. The head of the animal was placed towards the gantry. The imaging was done by Lotus-inVivo preclinical micro-CT scanner made in Iran. The technical factors of radiation and the CT scan method were as follows:

• Frame exposure time: 0.25 (s)
• Slice thickness: 0.1 (mm)
• Scan angle: 360
• Tube Voltage: 80 kVp
• Tube current: 95 µA

**Figure 3:**
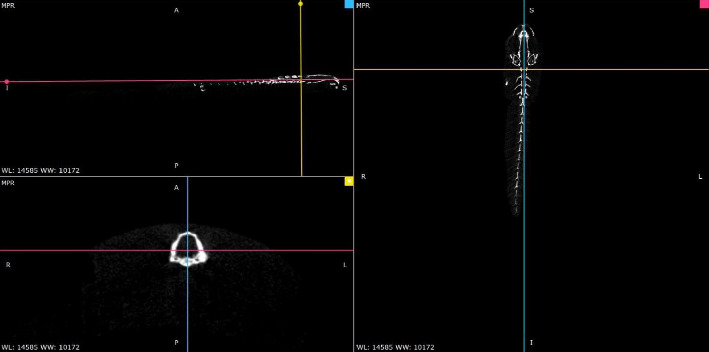
Screenshot of Radiant DICOM Viewer software settings.

One day after micro-CT scanning, the samples were transferred to their original habitat. The digital files of the samples, which were saved in DICOM format, were studied and morphologically evaluated by Radiant DICOM Viewer software (Figure 3).

## 3. Results

**Figure 4:**
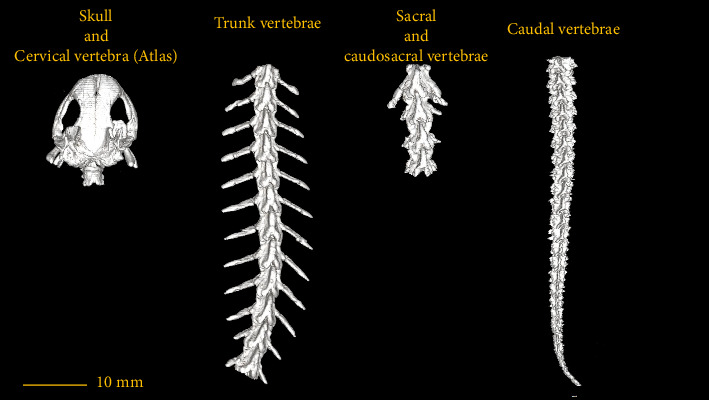
The volume rendering of micro-CT images of the *Neurergus kaiseri* newt skeletal indicate each segment of the vertebral column using Worthington and Wake classification [9].

**Figure 5:**
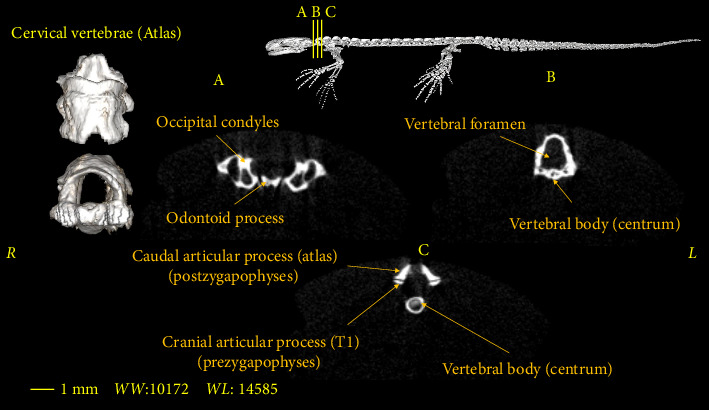
Two-dimensional and tree-dimensional micro-CT images of the cervical vertebra (atlas) of *Neurergus kaiseri*.

**Figure 6:**
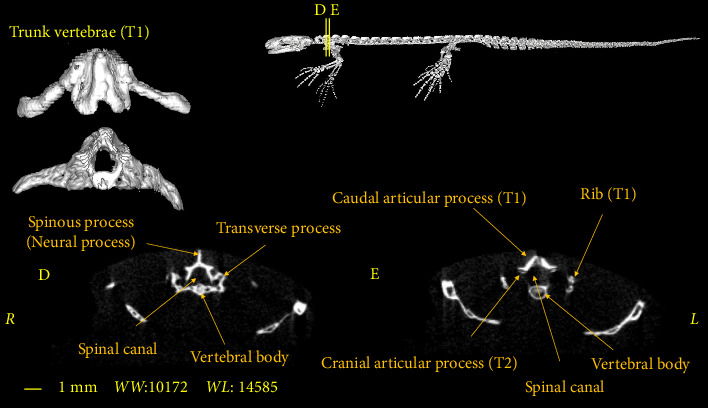
Two-dimensional and tree-dimensional micro-CT images of the first Trunk vertebra (T1) of *Neurergus kaiseri*.

**Figure 7:**
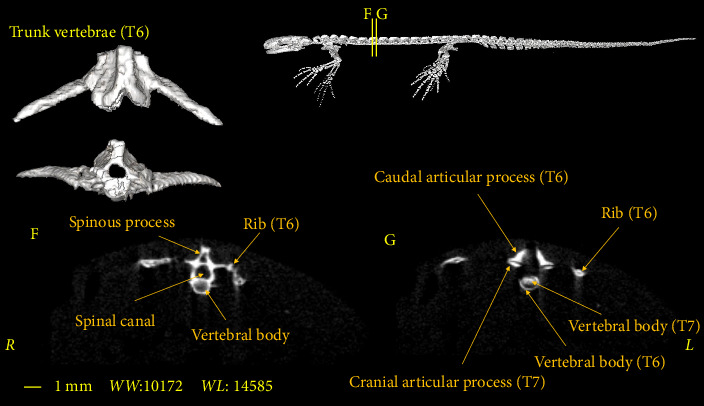
Two-dimensional and tree-dimensional micro-CT images of the sixth Trunk vertebra (T6) of *Neurergus kaiseri*.

**Figure 8:**
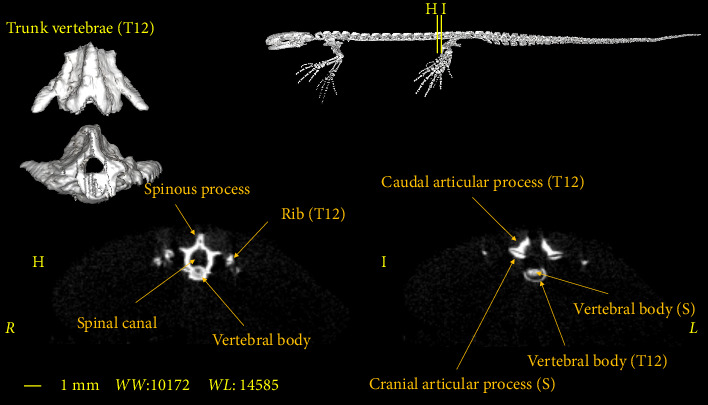
Two-dimensional and tree-dimensional micro-CT images of the twelfth Trunk vertebra (T12) of *Neurergus kaiseri*.

**Figure 9:**
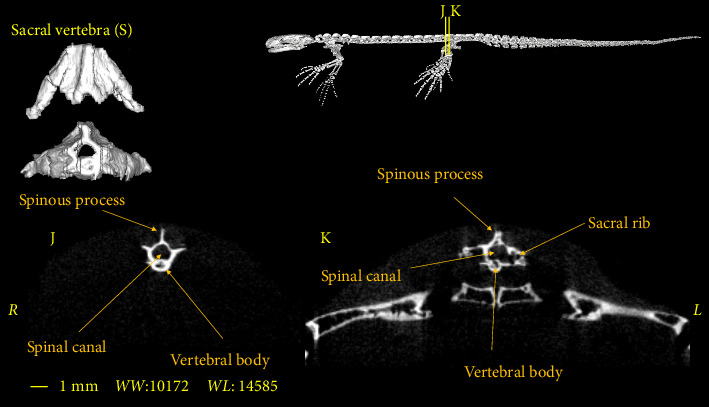
Two-dimensional and tree-dimensional micro-CT images of the sacral vertebra of *Neurergus kaiseri*.

**Figure 10:**
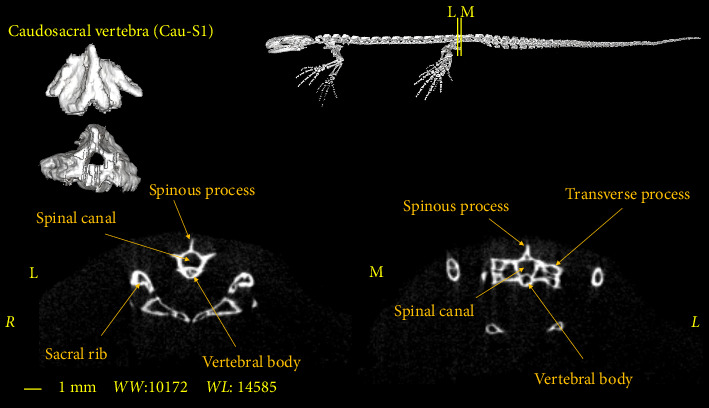
Two-dimensional and tree-dimensional micro-CT images of the first caudosacral vertebra of *Neurergus kaiseri*.

**Figure 11:**
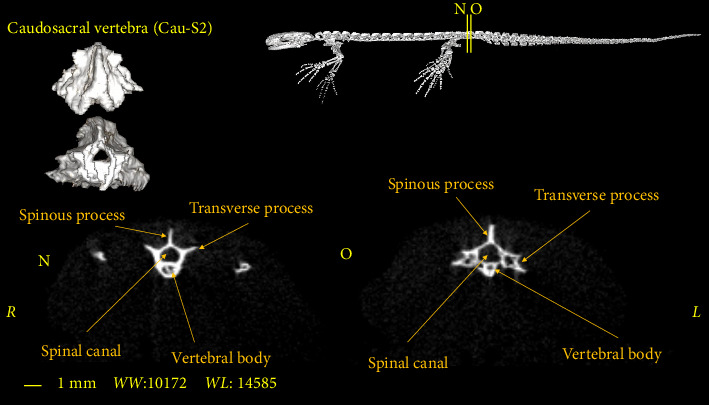
Two-dimensional and tree-dimensional micro-CT images of the second caudosacral vertebra of *Neurergus kaiseri*.

**Figure 12:**
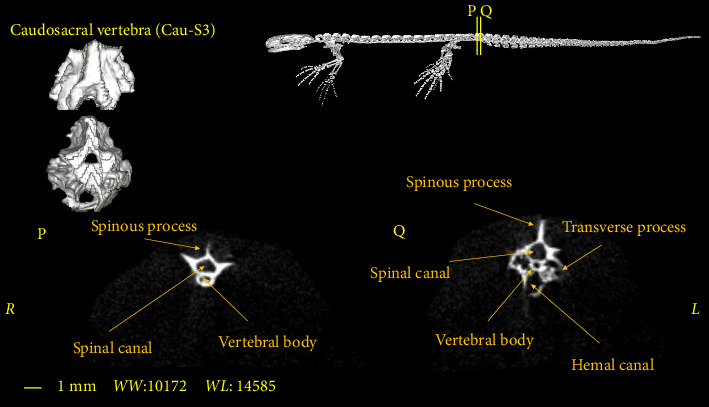
Two-dimensional and tree-dimensional micro-CT images of the third caudosacral vertebrae of *Neurergus kaiseri*.

**Figure 13:**
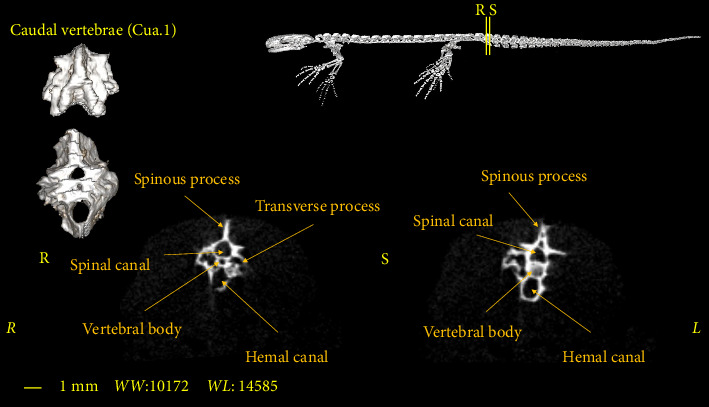
Two-dimensional and tree-dimensional micro-CT images of the first caudal vertebra of *Neurergus kaiseri*.

**Figure 14:**
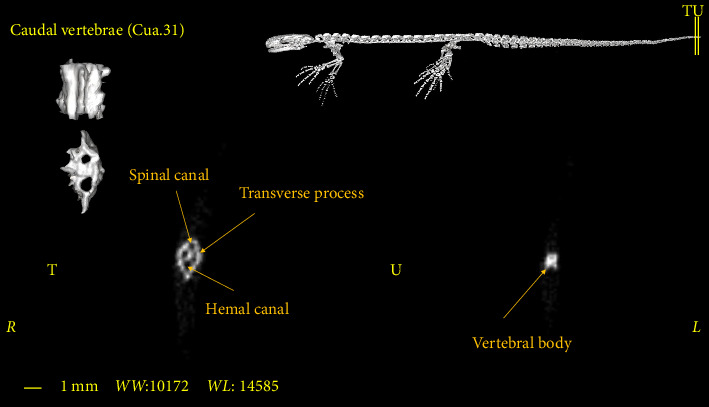
Two-dimensional and tree-dimensional micro-CT images of the last caudal vertebra of *Neurergus kaiseri*.

In this section, the vertebral column of the *Neurergus kaiseri* newt was studied. The different anatomical components of the vertebrae were named and the connections between these components were determined. Also, to show the exact position of these vertebrae in relation to each other and in more detail, three-dimensional reconstructions were made (Figure 4). The morphological characteristics of the vertebral column of the *Neurergus kaiseri* newt can be observed through two-dimensional and three-dimensional micro-CT images (Figures 5, 6, 7, 8, 9, 10, 11, 12, 13, 14). The *Neurergus kaiseri* newt has one cervical vertebra (atlas), 12 trunk vertebrae, one sacral vertebra, 3 caudosacral vertebrae, and 28 to 31 caudal vertebrae.

### 3.1. Cervical Vertebra (Atlas)

Atlas is wider in the cranial part than the caudal part in both the dorsal and ventral views, and it is generally shorter than the trunk vertebrae. Atlas has odontoid process in the cranial region that articulates with the occipital condyles of the skull and connects the head to the vertebral column (Figure 5(A)). The vertebral foramen, which passes from the cranial to the caudal parts, is almost triangular in shape at the beginning, which gradually becomes circular as it moves caudally (Figures 5(B) and 5(C)). Atlas lacks cranial articular processes (prezygapophyses), but caudal articular processes (postzygapophyses) are present. The caudal articular processes (postzygapophyses) articulate with the cranial articular processes (prezygapophyses) of the first trunk vertebra (Figure 5(C)).

### 3.2. Trunk Vertebrae

The *Neurergus kaiseri* newt has 12 trunk vertebrae (Figure 4). The spinal canal in the trunk vertebrae is often circular and as we move toward the caudal side, they are compressed dorsoventrally. Neural arches form the roof of the canal. These arches are interconnected and create the spinous process. In the cranial and caudal parts of all trunk vertebrae, the vertebral body has a round cross-section, which is slightly convex on the cranial surface of the vertebra and concave on the caudal surface of the vertebra. Each trunk vertebra has a pair of transverse processes that articulate with a pair of ribs and are directed toward the caudal side. All the ribs have one head and the ribs of the two or three last vertebrae have shorter sizes than the cranial vertebrae. The spinal canal passes through the dorsal surface of the vertebral body. Prezygapophyses and postzygapophyses articulate with the previous and subsequent vertebrae, respectively (Figures 6, 7, 8).

### 3.3. Sacral Vertebra

The sacral vertebra is similar to trunk vertebrae in terms of size and morphological characteristics. The spinal canal is mostly circular or oval in shape. In the cranial and caudal parts, the vertebral body has a round cross-section, which is slightly convex on the cranial surface and concave on the caudal surface of the vertebra. Prezygapophyses and postzygapophyses articulate with the last trunk vertebra and the first caudosacral vertebra, respectively. The sacral rib is significantly longer than the trunk ribs. In addition, the sacral rib curves ventrally and articulates distally with the pelvic girdle (Figure 9).

### 3.4. Caudosacral Vertebra

In all studied samples, the first three vertebrae, after the sacral vertebra, have the same dimensions and morphological characteristics as the trunk vertebrae. The first two vertebrae have no Hemal arch (Figures 10 and 11) and the third vertebra has the Hemal arch on the ventral side (Figure 12).

The first and second caudosacral vertebra are almost similar to the trunk vertebra, but there are also differences between them. The transverse process is smaller compared to the trunk and sacral vertebrae. The transverse process has a more vertical orientation towards the vertebral body compared to the previous vertebrae (Figures 10 and 11). The last caudosacral vertebra has the same characteristics as the previous vertebras and also has similarities with the caudal vertebrae, including the presence of Hemal arch (Figure 12).

### 3.5. Caudal Vertebrae

The caudal vertebrae are the last part of the vertebral column, all of which have a Hemal arch and often have a transverse process (Figure 13). The Hemal arch disappears only in the most extreme region of the vertebral column (Figure 14). These vertebrae have the longest spinous process among other vertebrae (Figure 13). The number of these vertebrae varies in different samples and in males and females. Therefore, it is not possible to consider a fixed number for these vertebrae in the *Neurergus kaiseri* newt. The adult specimens studied in this research had a maximum of 31 caudal vertebrae (Figure 4).

Female specimens had 28–31 caudal vertebrae, while the number of caudal vertebrae in male specimens was 30–31.

The average number of caudal vertebrae in male samples was 30.6 and in female samples were 29.6.

## 4. Discussion

In this study, the morphological characteristics of the vertebral column of the *Neurergus kaiseri* newt were investigated using the micro-CT method. It should be noted that the micro-CT scan is a better choice than radiography in bone studies, similar to our study. Since micro-CT scans do not have some of the drawbacks of radiography, such as image magnification and the limitation of taking images from certain angles, they are a better choice for morphometric studies. The present study, which was the first research to investigate the vertebral column in *Neurergus kaiseri* newt and its anatomical structure using micro-CT, can be used as a reference for other relevant studies.

Anatomical studies of the skeletal structures of an animal using modern diagnostic imaging techniques, such as a micro-CT, are the basis of many new scientific studies.

In a microscopic study by Khoshnamvand et al. on the Luristan newt (*Neurergus kaiseri*), it was reported that this species possesses 2 cervical vertebrae, 16 abdominal vertebrae, and 32 caudal vertebrae [15]. However, in the present study, micro-CT scan findings indicate the presence of 1 cervical vertebra, 12 trunk vertebrae, 1 sacral vertebra, 3 Caudo-sacral vertebrae, and a total of 28–31 vertebrae. In this study, by employing advanced micro-CT technology in both two-dimensional and three-dimensional imaging, we were able to discern detailed micro-anatomical structures that were previously underemphasized in earlier studies.

Morphological characteristics similar to this study have been investigated in other studies, such as Osteology of the Italian endemic spectacled salamanders [16], Osteology of mountain stream salamander from western China [17], and Osteological characteristics of the Setouchi salamander [18].

Consistent with the aforementioned studies, the vertebral column has five areas in the present study: the cervical region (atlas), trunk vertebrae, sacral vertebra, caudosacral vertebrae, and caudal vertebrae, respectively.

The number of cervical vertebrae in our research was similar to all the aforementioned studies and only the atlas was observed in them.

Similar to all the aforementioned studies, in our study, the atlas vertebra lacks ribs and transverse processes [16–18].

In our study, similar to all the aforementioned studies, the atlas vertebra has been reported to have an odontoid process. Despite of our research, Spinous process has been observed in all of the other research, that is the result of dorsal articulation of the neural arches. In our research, similar to the aforementioned research, the atlas vertebra has two pairs of caudal articular processes that articulates with the articular process of the first trunk vertebra [16–18].

There have been differences between the present research and the previous studies in terms of number of trunk vertebrae. In the osteology of mountain river salamanders in western China, it was found that they have 16 trunk vertebrae [17]. Satoshi salamander has 15–16 trunk vertebrae [18]. Native Italian salamanders had 12-13 trunk vertebrae [16]. Meanwhile, all samples of *Neurergus kaiseri* newts had 12 trunk vertebrae. All trunk vertebrae in the *Neurergus kaiseri* newt, such as native Italian newts, Satoshi newts, and western Chinese newts, had transverse processes and ribs, which extended along the transverse process [16–18]. Similar to findings of the study on native Italian newts, the spinal canal in the *Neurergus kaiseri* newt had a circular cross-section at the beginning of the trunk area and was compressed in the dorsoventral direction by moving towards the sacrum [16].

In all the aforementioned research, and also in the *Neurergus kaiseri* newt, there is a single sacral vertebra which has the same appearance characteristics as the trunk vertebrae, and it also has a transverse process and rib which is more prominent than the trunk vertebrae [16–18].

The number of caudosacral vertebrae in the *Neurergus kaiseri* newt is 3 vertebrae which is similar to that of native Italian newts, western Chinese newts, and Satoshi newts [16–18]. These vertebrae have been called caudosacral vertebrae by Worthington and Wake [9], although Francis considered these vertebrae as caudal vertebrae [19]. The first of which in all the mentioned studies as well as in the present study had characteristics similar to trunk vertebrae. Like native newts in Italy [16], the Hemal arch in the *Neurergus kaiseri* newt starts from the last caudosacral vertebra and extends to the end of the tail. In the Transverse process, the caudosacral vertebrae in the *Neurergus kaiseri* newt are smaller than the trunk and sacrum vertebrae and have a more vertical angle to the center of the vertebra, which is similar to native Italian newts [16].

The number of caudal vertebrae is different in various studies. Western Chinese Mountain salamander have a maximum of 33 caudal vertebrae [17]. This number has been reported to be more than 25 in Satoshi newt [18]. However, 28–31 caudal vertebrae were observed in the mountain *Neurergus kaiseri* newt. The caudal vertebrae of the mountain *Neurergus kaiseri* newt, like the Western Chinese Mountain salamander, have a transverse process [17], which is not present in the Satoshi newt [18]. In the native Italian newts, the western Western Chinese Mountain salamander, and the Satoshi newt, like the *Neurergus kaiseri* newt, the Hemal arch extends to the end of the caudal vertebrae [16–18].

## 5. Conclusion

In summary, the advanced micro-CT imaging techniques employed in this study have significantly enhanced our understanding of the skeletal structure of the Luristan newt (*Neurergus kaiseri*). The high-resolution two-dimensional and three-dimensional images enabled a meticulous examination of vertebral morphology, uncovering subtle transitional zones and distinct structural variations. Despite the inherent challenges of working with a limited number of specimens from an endangered species, our approach successfully balanced the need for rapid imaging to preserve specimen health with the demand for detailed anatomical data. Ultimately, these findings not only reinforce existing knowledge but also establish a robust framework for future comparative morphological studies and conservation strategies.

## Data Availability

The data that support the findings of this study are available from the corresponding author upon reasonable request.

## References

[1] Hickman C. P., Roberts L. S., Larson A., Anson H. I., Eisenhour D. J. (2006). *Integrated Principles of Zoology*.

[2] Bairbre O. M. (2005). Clinical Anatomy and Physiology of Exotic Species.

[3] George G. S. (1984). *Tempo and Mode in Evolution*.

[4] Akia F., Rastegar-Pouyani N., Faizi H. (2010). The Comparison of Cranial Osteology of Neurergus Microspilotus and *Salamandra infraimmaculata* Semenovi (Amphibia: Salamandridae). *Russian Journal of Herpetology*.

[5] Mobaraki A., Amiri M., Alvandi R. (2014). A Conservation Reassessment of the Critically Endangered, Lorestan Newt *Neurergus kaiseri* (Schmidt 1952) in Iran. *Amphibian and Reptile Conservation*.

[6] List IUCN Red (2004). The IUCN Red List of Threatened Species. *Di Sponí vel em*.

[7] Raymakers C. (2006). CITES, the Convention on International Trade in Endangered Species of Wild Fauna and Flora: Its Role in the Conservation of Acipenseriformes. *Journal of Applied Ichthyology*.

[8] Miller J. J. (2004). *Overview of the Salamandrid Genus Neurergus (Cope, 1862) Spotted Newts/Middle Eastern Newts*.

[9] Worthington R. D., Wake D. B. (1972). Patterns of Regional Variation in the Vertebral Column of Terrestrial Salamanders. *Journal of Morphology*.

[10] Kenneth V. K. (2006). *Vertebrates: Comparative Anatomy, Function, Evolution*.

[11] Ritman E. L. (2011). Current Status of Developments and Applications of Micro-CT. *Annual Review of Biomedical Engineering*.

[12] Dierick M., Van Loo D., Masschaele B. (2014). Recent Micro-CT Scanner Developments at UGCT. *Nuclear Instruments and Methods in Physics Research Section B: Beam Interactions With Materials and Atoms*.

[13] Khoshnamvand H., Malekian M., Keivany Y. (2018). Morphological Distinction and Sexual Dimorphism in Divergent Clades of *Neurergus kaiseri* (Amphibia: Salamandridae). *Basic and Applied Herpetology*.

[14] Meredith A., Redrobe S. (2002). *BSAVA Manual of Exotic Pets*.

[15] Khoshnamvand H., Malekian M., Keivany Y., Zamani-Faradonbe M., Amiri M. (2019). Descriptive Osteology of an Imperiled Amphibian, the Luristan Newt (*Neurergus kaiseri*, Amphibia: Salamandridae). *Acta Herpetologica*.

[16] Macaluso L., Villa A., Pitruzzella G. (2020). Osteology of the Italian Endemic Spectacled Salamanders, *Salamandrina* spp. (Amphibia, Urodela, Salamandridae): Selected Skeletal Elements for Palaeontological Investigations. *Journal of Morphology*.

[17] Jia J., Jiang J.-P., Zhang M.-H., Gao Ke-Q. (2019). Osteology of Batrachuperus Yenyuanensis (Urodela, Hynobiidae), a High-Altitude Mountain Stream Salamander From Western China. *PLoS One*.

[18] Hara S., Nishikawa K. (2022). Osteological Characteristics of the Setouchi Salamander Hynobius Setouchi (Urodela, Hynobiidae). *The Anatomical Record*.

[19] Eric Thomas Brazil Francis (1934). The Anatomy of the Salamander, by Eric TB Francis, With an Historical Introduction by Professor FJ Cole.

